# Mechanical biofilm disruption causes microbial and immunological shifts in periodontitis patients

**DOI:** 10.1038/s41598-021-89002-z

**Published:** 2021-05-07

**Authors:** W. Johnston, B. T. Rosier, A. Artacho, M. Paterson, K. Piela, C. Delaney, J. L. Brown, G. Ramage, A. Mira, S. Culshaw

**Affiliations:** 1 Oral Sciences, Glasgow Dental Hospital and School, School of Medicine, Dentistry and Nursing, College of Medical, Veterinary and Life Sciences, University of Glasgow, Glasgow, G12 8TA UK; 2 The Foundation for the Promotion of Health and Biomedical Research (FISABIO), Avda. de Catalunya, 21, 46020 Valencia, Spain; 3 Division of Dentistry, Medical University of Lodz, Lodz, Poland; 4 Centre for Epidemiology and Public Health, Monforte de Lemos, 5, ES-28029, Madrid, Spain

**Keywords:** Periodontitis, Biofilms, Cytokines, Clinical microbiology, Biomarkers

## Abstract

Periodontitis is characterized by subgingival biofilm dysbiosis, inflammation and tissue destruction. Current treatment involves mechanical biofilm disruption known as non-surgical periodontal therapy (NSPT). This study sought to characterise the impact of treatment on microbial diversity and overall community, and the parallel impact on host inflammation in the oral cavity. Fourty-two periodontitis patients were included in this study, with periodontal clinical parameters, subgingival plaque and saliva samples collected at baseline and 90 days after treatment. Salivary cytokines were quantified, and subgingival plaque was analysed using 16S rRNA sequencing. After treatment, there were marked health-associated alterations in microbial composition and diversity, including differential abundance of 42 genera and 61 species. These changes were accompanied by substantial clinical improvement (pockets ≥ 5 mm, 27.50% to 9.00%, *p* < 0.001) and a decrease in salivary IL-1β (*p* < 0.001)—a putative marker of periodontal inflammation. Despite significant reductions in disease associated anaerobes, several genera (*Fusobacterium, Prevotella, Tanenerella, Treponema*) remained present and formed a distinct subnetwork associated with residual disease. Collectively, this study shows that current periodontal treatment results in partial restoration of a healthy microbial ecosystem, but features of biofilm dysbiosis and host inflammation remain in some patients, which were surprisingly independent of clinical response.

## Introduction

Periodontitis (PD) is a highly prevalent, biofilm-mediated chronic oral inflammatory condition affecting 50% of the population, with 10% suffering from severe disease^1,2^. PD arises via the formation of dysbiotic subgingival plaque biofilms, which perpetuate an aggravated immune response within the gingival tissue^3^. In turn, this leads to a continuous cycle of host-bacterial interplay, whereby inflammation facilitates greater dysbiosis of the microbiome, and vice-versa^4^. If left untreated, this immune response leads to irreversible damage to the periodontal ligament and alveolar bone, ultimately resulting in tooth loss in susceptible individuals. Emerging evidence suggests that the consequences of PD may extend beyond the oral cavity, with previous work suggesting an association between periodontitis and risk of diabetes^5,6^, rheumatoid arthritis^7–9^, atherosclerosis^10,11^, hypertension^12,13^ and Alzheimer’s disease^14^. Given the nutritional and psychosocial consequences of tooth loss and the links between PD and systemic disease, restoring periodontal health (PH) is essential to both oral and general health. Current treatments are resource intensive, time consuming and often only partially successful. Therefore, there is a need to understand the microbial and host immune response to further optimise treatment outcomes.

Several studies have consistently documented a unique bacterial signature of the subgingival plaque in PD, with Socransky’s seminal findings of distinct complexes—each designated a colour—desbribing related groups of bacteria based on their association with health and disease (red, orange, green, purple, blue, yellow)^15^. Of note, the ‘red complex’ consisting of *Porphyromonas gingivalis, Tannerella forsythia* and *Treponema denticola* were found to strongly relate to PD severity. More recently, advances in molecular approaches have revealed that the diversity of the subgingival plaque is higher than previously assumed^16–20^. Whilst the ‘red complex’ relationship with disease remains^21^, large-scale analyses have revealed dysbiosis of the entire plaque community, associated with increased microbial diversity and anaerobic bacteria^16,17^. In line with these findings, a recent systematic review documented several new species associated with PD such as *Filifactor alocis, Fretibacterium fastidiosum* and *Treponema vincentii*^22^.

Chronic inflammation in response to dysbiotic subgingival plaque biofilms is a consistent feature of PD and escape of locally generated inflammatory mediators into the circulation has been proposed as a putative link between oral and systemic health^23^. Pro-inflammatory cytokines in saliva have been implicated as potential biomarkers for PD, with independent cross-sectional studies showing higher levels of several cytokines in PD compared with health^24,25^. Yet, this does not appear to be consistent across independent studies and patient cohorts. For example, Ebersole et al*.,* found that salivary Interleukin-1β and Interleukin-6 were elevated in 101 PD patients compared with 65 healthy controls^24^. In contrast, Teles et al*.,* found no significant differences when assessing both cytokines in the saliva of 74 PD patients and 44 healthy controls^26^. Hence, characterising how cytokines respond to improvements in clinical status may provide a critical insight into their role in PD.

Given the previous identification of host and microbial markers associated with PD, we sought to evaluate how these markers reflect disease severity and change following treatment and improvements in clinical status. Non-surgical periodontal therapy (NSPT) represents the first line of treatment for the majority of PD patients and involves physical debridement of biofilms from beneath the gum-line using hand or ultrasonic scaling instruments, inducing widespread—although not total—clinical improvement^27, 28^. To prevent disease recurrence and potential systemic complications, it is crucial to understand whether physical biofilm disruption is sufficient to reduce local inflammation and microbial dysbiosis. Salivary cytokines and microbiome have been well characterised in cross sectional studies comparing health and disease^16,17,24,26^; however, there is limited understanding of changes in response to treatment. In this study, we aimed to investigate the subgingival plaque microbiome and salivary inflammatory cytokines—in relation to baseline disease severity, and longitudinally following NSPT.

## Methodology

### Study population, treatment and clinical examination

This longitudinal study was conducted in accordance with the Declaration of Helsinki (2013), with ethical approval (London-Stanmore Research Ethics Committee, Reference: 14/LO/2064). Eligible patients were recruited from referrals to Glasgow Dental Hospital. Periodontitis was defined as probing pocket depths ≥ 5 mm on 2 or more teeth at non-adjacent sites excluding third molars^29^. Additional inclusion criteria included written informed consent, and male or female ≥ 18 years of age. Exclusion criteria included patients with diabetes, rheumatoid arthritis, known or suspected risk of tuberculosis, hepatitis B or HIV infections, or history of bleeding diathesis. In total, 45 patients were recruited and provided written consent to take part in the study. Three patients were subsequently excluded due to a new diagnosis of a systemic disease during the trial period. Therefore, 42 patients were included for analysis.

**Figure 1:**
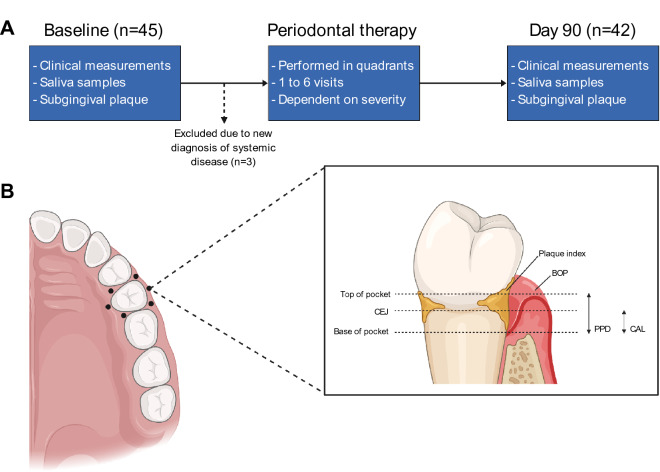
Outlining study protocol (**A**) and collection of periodontal clinical measurements (**B**). Six sites around each tooth were measured for plaque index, BOP, PPD and CAL. CEJ; Cementoenamel junction, BOP; Bleeding on probing, PPD; Probing pocket depth, CAL; Clinical attachment loss. Image created using BioRender.

NSPT was delivered by a single experienced dental hygienist, who was calibrated with experienced periodontists. Patients returned for varying numbers of treatment visits (between 1–6) depending on disease severity and preference for treatment scheduling. Periodontal parameters were assessed before any treatment visits (baseline) and 90-days following the last treatment visit (day 90) (Fig. 1A). Periodontal parameters were assessed at six sites per tooth, including full-mouth plaque scores^30^ and full-mouth bleeding scores^31^ (Fig. 1B). Periodontal pocket depths (PPD), location of the gingival margin (LGM) and clinical attachment level (CAL) were recorded to the nearest millimetre per site and divided by the total number of sites to give average full-mouth scores. The periodontal inflamed surface area (PISA) was calculated as described previously^32^.

### Sample collection and processing

Subgingival plaque and saliva were collected at baseline and day 90. At baseline, subgingival plaque was collected from the deepest pocket in each quadrant using a curette and placed into an Eppendorf tube containing 500uL sterile phosphate buffered saline (Sigma Aldrich, Gillingham, UK). Bacterial cells were harvested via centrifugation (13,500 RPM for 10-min) and supernatant was discarded. One site was selected from each patient for sequencing, and samples from the same site were sequenced before and after treatment to ensure accurate longitudinal analysis. Samples collected from the upper right quadrant were preferentially selected (n = 35), unless unable to sample the same site at day 90. In this case, pre- and post-treatment samples from a different quadrant were selected; upper left (n = 1), lower right (n = 3), lower left (n = 3). Saliva was collected using the passive drool method and was clarified via centrifugation (13,000 RPM for 5-min) as described previously^33^. All samples were transferred to adjacent laboratories and processed immediately. Samples were stored at -80 °C until analysis.

### Analysis of salivary cytokines

Concentrations of salivary tumour necrosis factor α (TNFα), interleukin-6 (IL-6) and interleukin-1β (IL-1β) were determined using enzyme-linked immunosorbent assay (ELISA) kits (Thermofisher, Loughborough, UK). Salivary interleukin-8 (IL-8) was quantified using DuoSet ELISA kit (Biotechne—R&D Systems, Abingdon, UK). Salivary interleukin-17A (IL-17A) was quantified using high-sensitivity ProQuantum immunoassays, performed using a StepOne plus real-time PCR analyser (Thermofisher, Loughborough, UK). Samples were diluted 1:2 for IL-1β, TNFα and IL-6 assays, 1:3 for IL-8 assays and 1:5 for IL-17A assays, as per manufacturers instructions. One patient had limited sample volume and was not included in any salivary analysis (n = 41 for salivary cytokines). The limit of detection (LOD) for cytokines was IL-6: 0.04 pg/mL, IL-1β: 1.35 pg/mL, IL-8: 6.78 pg/mL, TNFα: 0.14 pg/mL and IL-17A: 2.75 pg/mL. IL-6, IL-1β and IL-8 were detected in all samples. TNFα was < LOD in 3 samples (1 at baseline, 2 at day 90) and IL-17A was < LOD in 12 samples (10 at baseline, 2 at day 90). For statistical analysis, samples < LOD were assigned LOD/2.

### Bacterial 16S rRNA sequencing

DNA was extracted from subgingival samples (baseline and day 90) using the MagNA Pure LC DNA isolation kit (Roche Diagnostics, Mannheim, Germany) with the addition of a chemical lysis step with an enzymatic cocktail containing lysozyme, mutanolysin and lysostaphin, following Rosier et al*.* 2020^34^. DNA concentrations were measured using a QubitTM 3 Fluorometer (Thermofisher, Waltham, Massachusetts, USA), and an Illumina amplicon library was prepared following the 16S rRNA gene Metagenomic Sequencing library preparation Illumina protocol (Part #15,044,223 Rev. A). The primer sequences used in this protocol were; *Illumina_16S_341F* (TCGTCGGCAGCGTCAGATGTGTATAAGAGACAGCCTACGGGNGGCWGCAG) and *Illumina_16S_805R* (GTCTCGTGGGCTCGGAGATGTGTATAAGAGACAGGACTACHVGGGTATCTAATCC) which target the 16S V3 and V4 region. Following amplification, DNA was sequenced with an Illumina MiSeq Sequencer according to manufacturer’s instructions using the 2 × 300 base paired-ends protocol. For taxonomic classification, an amplicon sequence variant (ASV) table was obtained using the DADA2 pipeline in R^35^. Taxonomy was assigned by comparison to the SILVA database^36^, where the naive Bayesian classifier was used to assign sequences at the genus- and species-level.

### Data analysis

Data was initially collected into SPSS (v26, IBM). Following collection, longitudinal analysis of clinical characteristics and salivary cytokines was performed using GraphPad PRISM (v8) using Wilcoxon signed rank tests. Correlation analysis was conducted using Spearman-rho (non-parametric) or Pearson’s (parametric) depending on data distribution, which was determined by visual inspection of histograms.

For analysis of the subgingival plaque microbiome, R programming language (v3.4 +) was used for statistical computing^37^. Alpha-diversity indexes (Shannon, ACE, Chao1) were calculated at species-level rarefying to 9000 reads using the Vegan library in R^38^, and longitudinal differences were assessed using paired t-tests in GraphPad PRISM (v.8).

Relative abundances were calculated using the total sum scaling (TSS) method. Only genera and species with a minimal signal of detection were included in microbiome statistical analysis. Specifically, a species or genus was included if it was present in 50% of the samples from at least one of the two groups (relevant for Wilcoxon tests) or from the given group under study (relevant for association networks and Spearman’s correlations) with an abundance superior to five times the smallest percentage above zero. For univariate analyses, Wilcoxon signed rank tests (i.e., wilcox.test function of stats library of R) were performed to test the differences in genera and species between baseline and day 90, corrected for multiple comparisons using the Benjamini–Hochberg false discovery rate (FDR) of 5%^39^. Thus, only adjusted p-values are reported for taxonomic comparisons. To compare the abundance of known health and disease-associated organisms, species were grouped according the colour complexes outlined by Socransky et al^15^. The grouping represents the sum of relevant species, outlined in Supplementary Table 2.

For association networks, pairwise associations were computed between genera and species based on a multivariant approach described by González et al.^40^, which was implemented in the 'mixOmics' R package^41^. In short, association between genera and species are obtained based on their projection onto a correlation circle plot derived from a principal component analysis. In the association networks, only negative associations below -0.4 and positive associations above 0.4 between genera are shown. The network graphs were obtained with the R ‘ggraph’ package^42^. To complement the association networks, correlations between genera and species were determined with Spearman's rho, along with associated p-value using the cor.test function.

For multivariant analysis, an Adonis test (Permutational Multivariate Analysis of Variance Using Distance Matrices), provided by the Vegan library of R^38^, was used to compare the overall composition between groups. To visualize groups and their differences in a two-dimensional map, we computed constrained principal components via constrained correspondence analysis (CCA) which is also part of Vegan library^38^. Principal coordinate analysis (PCoA) was conducted using MicrobiomeAnalyst^43^, with Bray–Curtis similarity calculated using PAST software (v4.01)^44^. All graphs were assembled using GraphPad PRISM (v8) or R^37^.

## Results

### Study population and clinical parameters

Patients with periodontitis who required specialist periodontal treatment were recruited (Fig. 1A). The average age of patients included in the analysis was 50 years old, with 12 males and 30 females (Supplementary Table 1). Following treatment, there was a marked improvement in all periodontal clinical parameters (*p* < 0.001 for all, Table 1). Compared with full-mouth parameters (average of all teeth), there was higher disease severity at baseline and greater clinical improvement following treatment in sites from which subgingival plaque samples were collected (PPD and CAL, both *p* < 0.001, Table 1).

**Table 1:** Comparison of periodontal parameters at baseline and day 90 (n = 42).

Variable	Baseline (n = 42)		Day 90 (n = 42)		*p*-value^§^
**Full-mouth**					
FMPS (%)	71.00 (46.75, 76.50)		12.0 (8.75, 29.25)		< 0.001
FMBS (%)	61.00 (32.75, 76.25)		8.00 (3.75, 15.25)		< 0.001
PPD (mm)	3.59 (3.08, 4.16)		2.78 (2.56, 3.02)		< 0.001
PPD ≥ 5 mm (%)	27.50 (18.75, 41.75)		9.00 (5.75, 12.25)		< 0.001
CAL (mm)	4.43 (3.73, 5.33)		3.72 (3.18, 4.31)		< 0.001
PISA (mm^2^)	1224.60 (754.80, 1687.08)		138.85 (65.52, 293.88)		< 0.001
**Sites from which plaque sample collected**					
PPD (mm)	7.00 (6.00, 8.00)		4.00 (4.00, 5.00)		< 0.001
CAL (mm)	8.00 (7.00, 9.00)		6.00 (5.00, 7.00)		< 0.001

Data represent full-mouth and the site from which subgingival plaque was harvested. Data are displayed as medians (Q1, Q3). FMPS; full-mouth plaque score, FMBS; full-mouth bleeding score, PPD; probing pocket depth, CAL; clinical attachment level, PISA; periodontal inflamed surface area.

^§^*p*-values refer to Wilcoxon signed rank test.

### The microbiome following periodontal treatment

**Figure 2:**
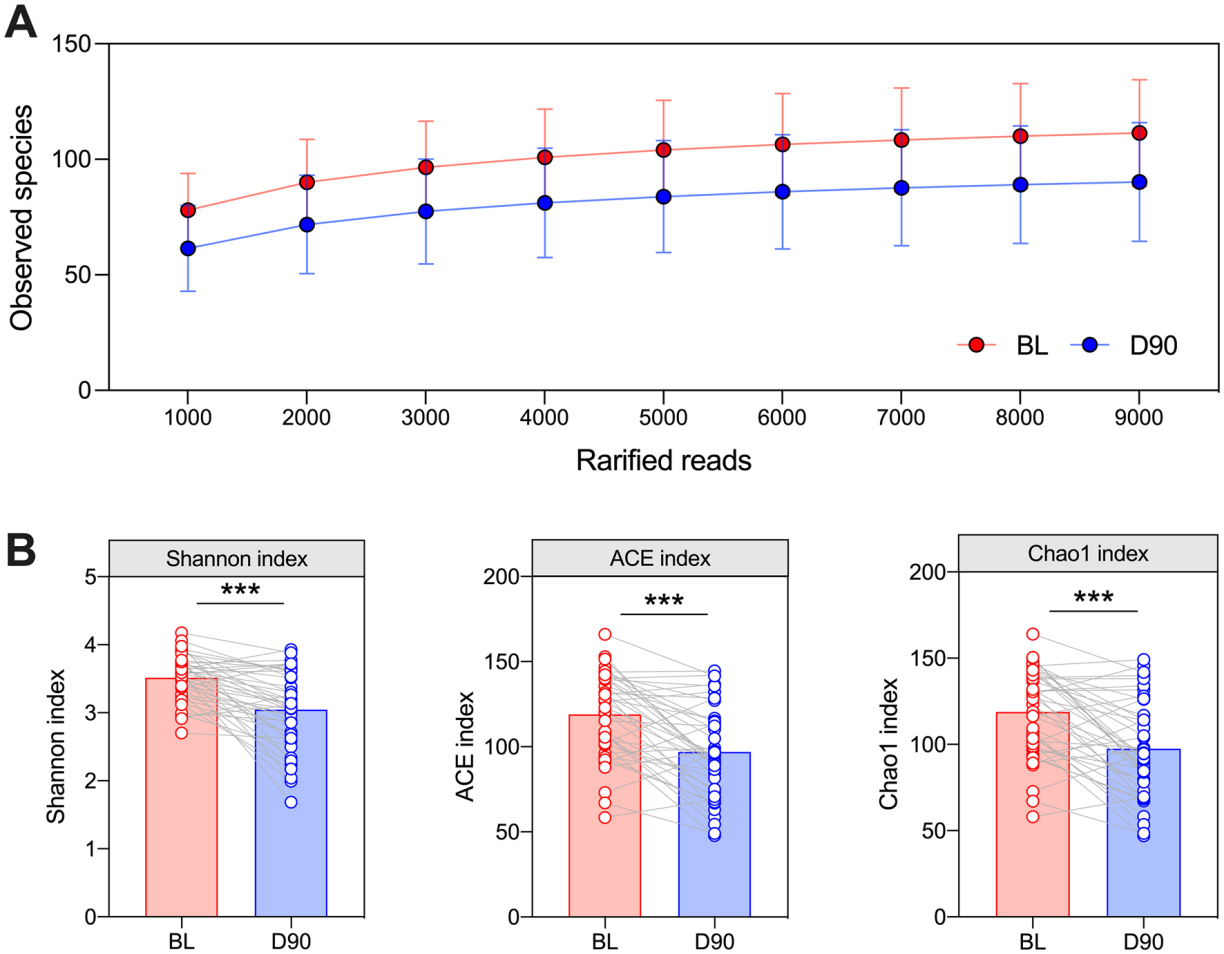
Alpha-diversity of subgingival plaque samples. Rarefaction curves for observed species at baseline (red line) and day-90 (blue line) (**A**). Comparing Shannon, ACE and Chao1 indexes before and after treatment at species-level rarefied to 9000 reads (**B**). Statistics are paired t-test, ***p < 0.001. n = 42. Bars represent mean with individual values shown.

Sequencing of subgingival plaque samples yielded 4745 ASVs across 84 samples, with an average of 90,830 reads per sample (ranging from 35,372 to 343,259 reads). After removing singletons, these ASVs were classified into 206 genera and 379 species. Rarefaction curves suggested a reliable estimate of taxonomic diversity was feasible rarefying to 9,000 reads per sample (Fig. 2A). Following treatment, there was a marked reduction in subgingival plaque taxonomic diversity, independent of method of evaluation (*p* < 0.001 for all, Fig. 2B).

**Figure 3:**
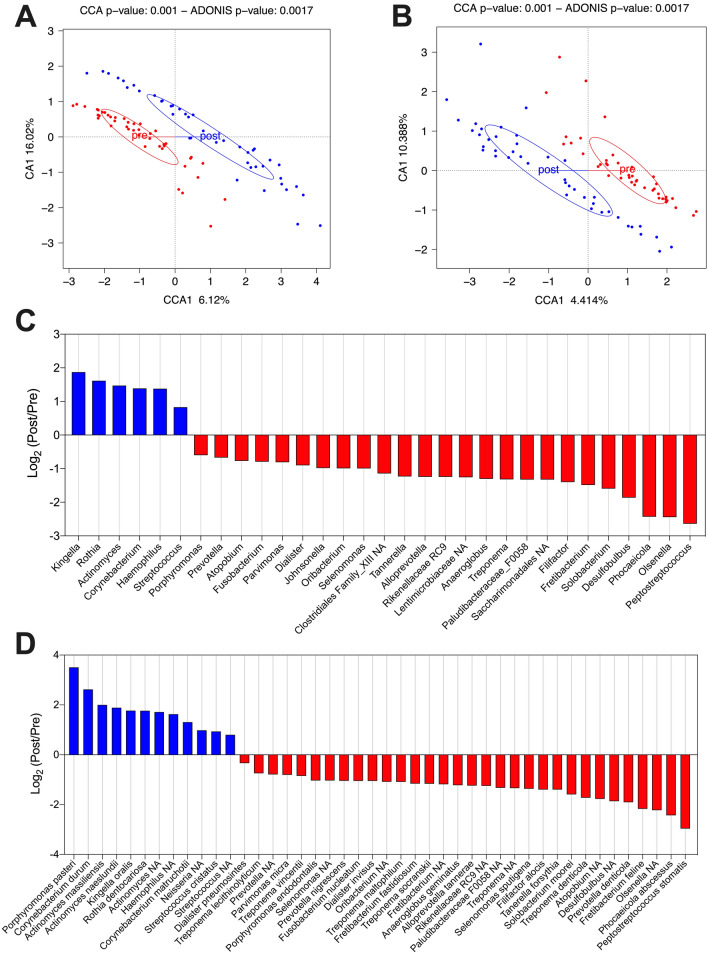
Assessing longitudinal changes in the composition of sub-gingival plaque samples following treatment. Canonical Correspondence Analysis (CCA) comparing baseline (red) and day-90 (blue) samples at genus- and species-level (**A** and **B** respectively). Log_2_ fold change of all significant genera (**C**) and species (**D**) with average abundance > 0.15% at either timepoint. Positive values (blue bars) indicate organisms which increased significantly following treatment, negative values (red bars) indicate organisms which decreased significantly following treatment. Wilcoxon signed rank test, corrected using Benjamini–Hochberg false discovery rate (5%). All plotted organisms *p*(adjusted) < 0.05. n = 42. NA indicates no classification was obtained at genus- or species-level.

Along with shifts in microbial diversity, treatment resulted in clear alterations to the microbiota composition, identified using CCA analysis at genus—(Fig. 3A) and species-level (Fig. 3B). Evaluation of genera and species which differed significantly revealed changes in 42 genera and 61 species comparing baseline and day 90 samples (Supplementary Fig. 1). Of the 42 genera, 34 decreased whilst 8 increased in abundance; at species level 46 species decreased and 15 species increased in abundance following treatment (Supplementary dataset 1). To identify genera and species contributing most to the compositional alterations, a further abundance filter (average abundance > 0.15% at either timepoint) was applied, and demonstrated differential abundance of 31 genera and 44 species. Of the 31 genera, 25 decreased whilst 6 increased in abundance (Fig. 3C). A similar trend was observed at species-level, with 32 species decreasing and 12 species increasing in abundance following treatment (Fig. 3D).

Overall there was a clear trend whereby the abundance of strict anaerobic species decreased following NSPT, and facultative anaerobic or aerobic bacteria increased. Interestingly at species-level the largest fold-increase was found for *Porphyromonas pasteri* (p[adjusted] = 0.02) belonging to a conventionally disease-associated genus, though it remained at relatively low abundance (0.6% of total, Supplementary Fig. 1).

### Changes in health and disease-associated organisms

**Figure 4:**
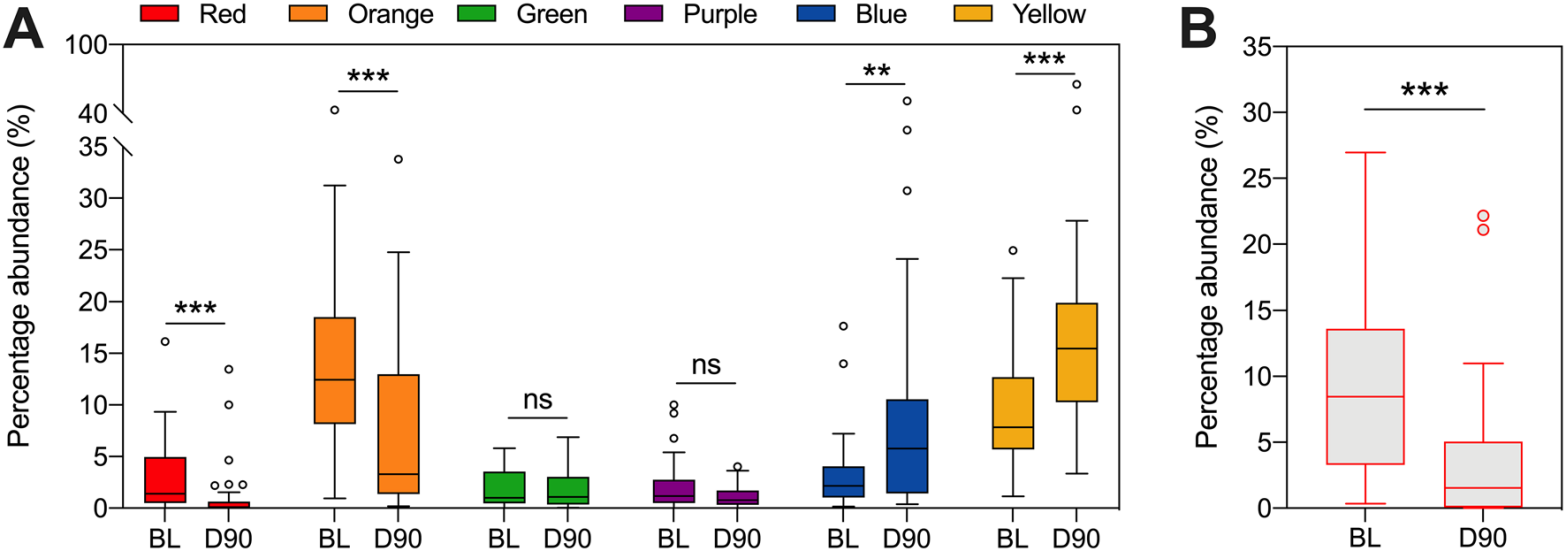
Comparing the abundance of health and disease associated species in the subgingival plaque. Species detected by 16S rRNA sequencing were grouped in the Socransky (1998) red, orange, green, purple, blue and yellow complexes (**A**). Comparing the sum of ‘novel’ species associated with disease identified by Perez-Chaparro (2014) (**B**). Species included in figure B are; *Treponema medium, Peptostreptococcus stomatis, Prevotella denticola, Mogibacterium timidum, Filifactor alocis, Selenomonas sputigena, Alloprevotella tannerae, Anaeroglobus geminatus, Fretibacterium fastidiosum, Porphyromonas endodontalis, Treponema vincentii, Treponema lecithinolyticum and Dialister pneumosintes*. Tukey box plots of all individuals (N = 42) are shown. Wilcoxon signed rank test where ***p < 0.001, *p < 0.05, ns means no significant difference. *P*-values were adjusted using FDR (5%).

Historically, Socransky et al*.*^15^ identified distinct complexes and assigned each a colour (red, orange, green, purple, blue and yellow), depending on their strength of association with disease. Red and orange complexes contain groups of disease-associated organisms, whilst the green, purple, blue and yellow complexes contain species more associated with PH (Supplementary Table 2). Following treatment, there were significant reductions in the abundance of the red and orange complexes (*p* < 0.001 for both). No difference was observed in green and purple complexes, whilst significant increases were found in the blue (*p* < 0.01) and yellow (*p* < 0.001) complexes at day 90 (Fig. 4A). A recent review by Perez-Chaparro et al*.,* identified a more complex community of disease associated bacteria^22^, and there was a similar reduction in the abundance of these species following treatment when grouped together (*p* < 0.001, Fig. 4B). Significant decreases were found in the majority of individual species within this group (*p* < 0.05, Wilcoxon signed rank tests), with the exception of *Treponema medium* and *Mogibacterium timidum*, which were present only at very low abundance.

### Genera abundance and association networks

Along with compositional changes (Supplementary Fig. 2), analysis of bacterial association networks revealed a shift in the overall community structure. Network analysis was performed using correlation circle plots (Supplementary Fig. 3), and highlighted distinct clustering of individual genera and species at each timepoint. Most associations shown between genera (93.2% at baseline, 90.0% at day 90) and species (88.3% at baseline, 90.3% at day 90) were supported by significant Spearman-Rho correlations (*p*(adjusted) < 0.05, Supplementary dataset 1).

**Figure 5:**
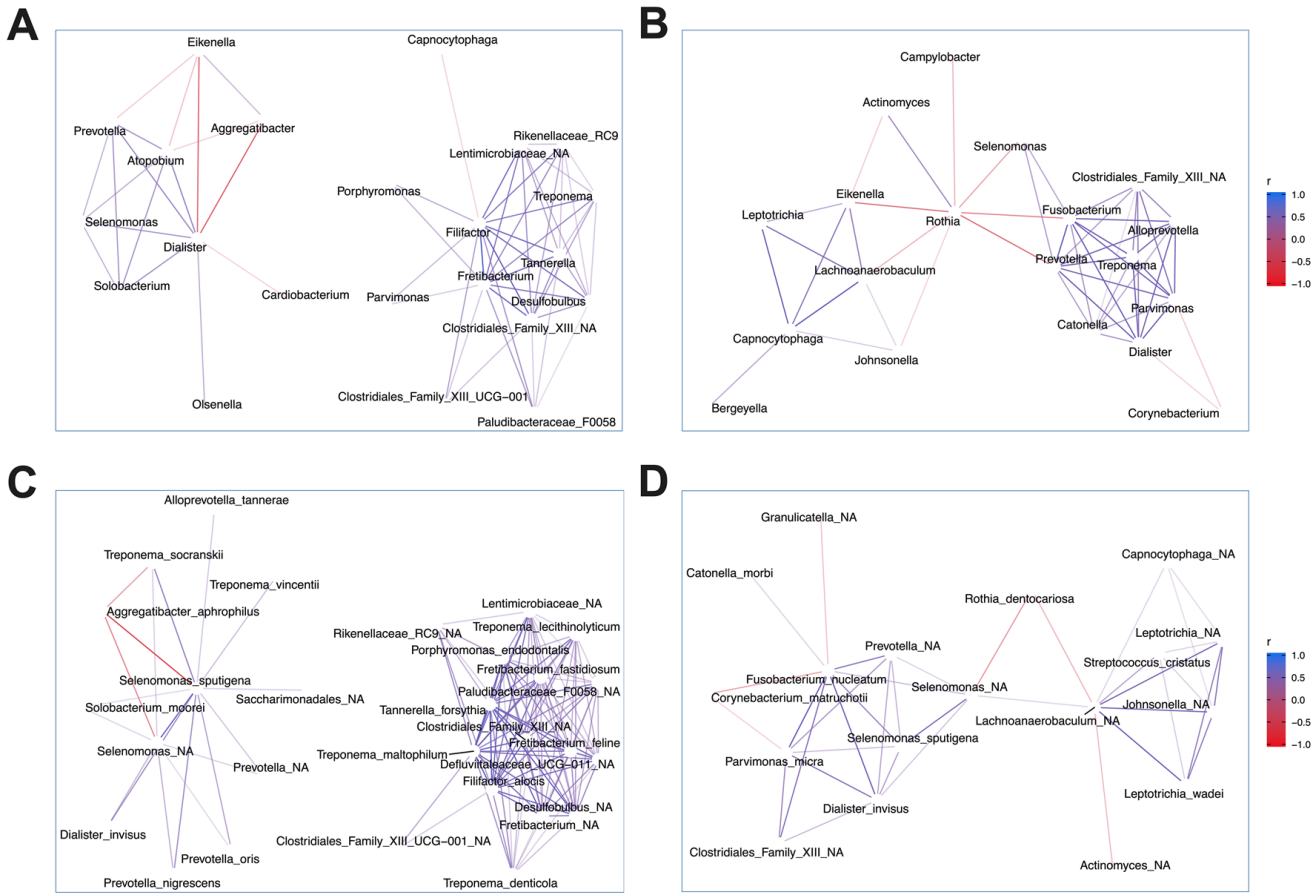
Bacterial association networks. Association networks were constructed between abundant genera and species based on correlation circle plots derived from  principal component analysis at each timepoint. (**A**) Genus-level network at baseline, (**B**) genus-level network at day 90, (**C**) species-level network at baseline, (**D**) species-level network at day 90. Only associations <  − 0.4 (negative associations) and > 0.4 (positive associations) are displayed.

At baseline there were two disease-associated clusters consisting of anaerobic genera (Fig. 5A). The first pre-treatment network consisted of *Prevotella*, *Selenomonas*, *Dialister*, *Solobacterium*, *Olsenella* and *Atopobium*, whilst *Eikenella* and *Aggregatibacter* showed negative associations with some of these genera (Fig. 5A, red lines). Within the second cluster, *Filifactor* and *Fretibacterium* were central players, both correlating positively with all three red-complex genera (i.e., *Porphyromonas*, *Treponema*, *Tannerella)*. After treatment (Fig. 5B), a new disease-associated network was observed consisting of *Treponema*, *Prevotella, Parvimonas, Fusobacterium*, *Alloprevotella*, *Selenomonas*, *Dialister*, and *Catonella*. Importantly, *Rothia* showed negative associations with *Selenomonas*, *Fusobacterium*, and *Prevotella* of this cluster, whilst a positive correlation was found between *Rothia* and *Actinomyces*.

Similar results were observed at species-level, where *Filifactor alocis, Fretibacterium fastidiusum* and *Fretibacterium feline* were central in a closely associated network of anaerobic bacteria, forming strong connections to various PD-associated organisms at baseline (Fig. 5C). Following treatment (Fig. 5D), *Rothia dentocariosa* negatively associated with unclassified *Selenomonas* sp. and *Lachnoanaerobaculum* sp., whilst *Corynebacterium matruchottii* showed negative associations with *Fusobacterium nulceatum* and *Parvimonas micra,* two orange-complex species.

### Microbiome, disease severity and response to treatment

To investigate the relationship between the microbiota and clinical disease severity, Spearman-Rho correlations were performed between the pocket depth of sampled teeth, individual genera/species abundance and alpha-diversity indexes. No associations were observed between the site pocket depth and the abundance of any genera or species. However, when considering the disease severity of the entire tooth (average of the 6 sites), several positive and negative associations were noted. At baseline, there was a weak positive correlation between tooth pocket depth and alpha-diversity indexes, particularly the Shannon index (r = 0.403, *p* = 0.008), whilst no association was found with the abundance of any individual genera or species. At day 90, there was no association with the Shannon index (Supplementary Table 3). Instead, weak positive correlations were observed between tooth PPD and strict anaerobic genera (*Treponema*, *Prevotella*, *Parvimonas*, *Alloprevotella*, *Dialister* and unclassified *Clostridiales Family XIII*), whilst negative correlations were observed for facultative anaerobes (*Corynebacterium*, *Granulicatella* and *Kingella*). Interestingly, all of the genera which positively correlated with tooth pocket depth were also key members of the remaining disease-associated network identified at day 90 (Fig. 5C). In contrast, *Corynebacterium*, which negatively correlated with pocket depth, showed negative associations with two of these genera (*Dialister* and *Parvimonas*).

**Figure 6:**
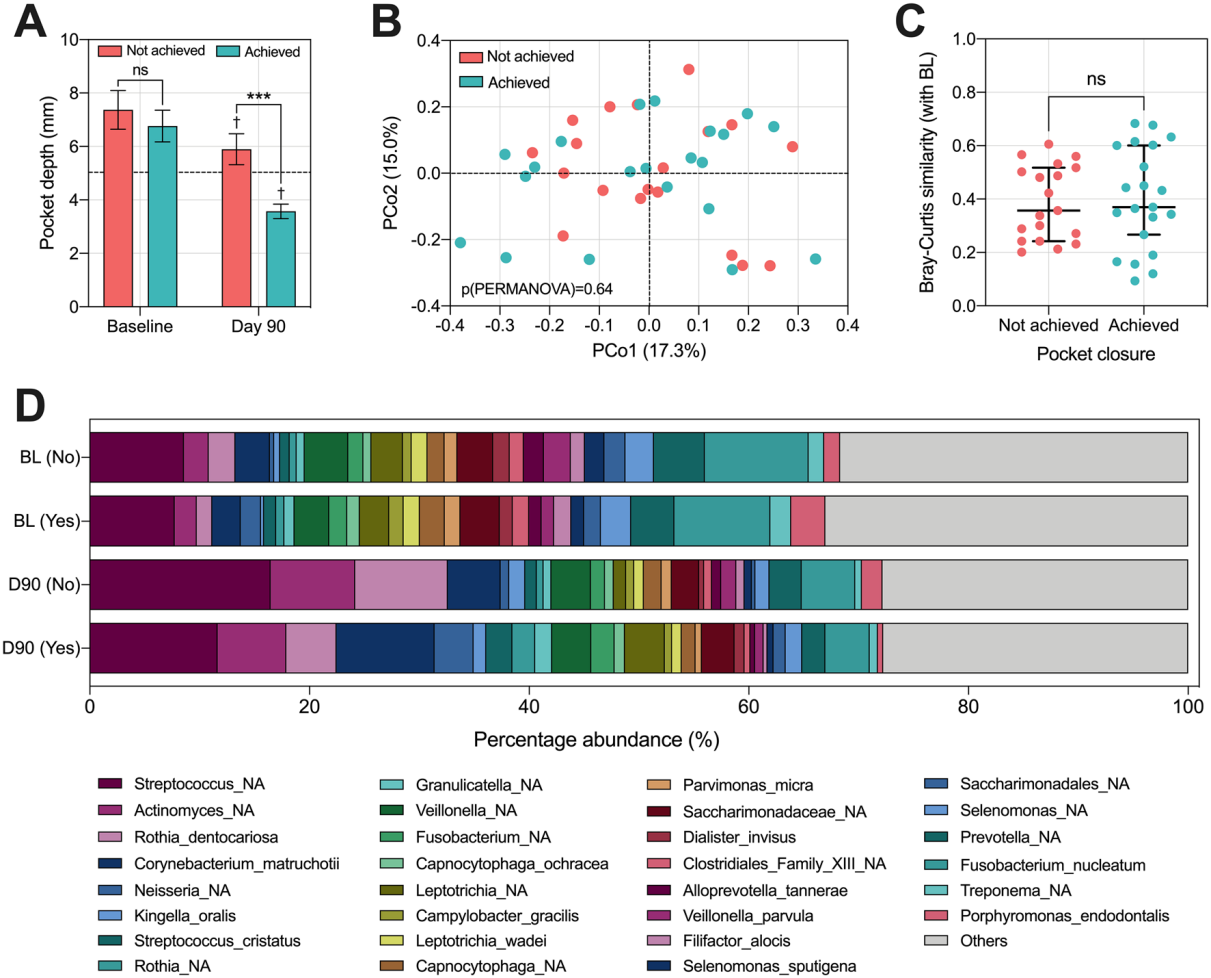
Investigating the subgingival plaque microbiome in relation to treatment response. (**A**) Comparing the pocket depth of sampled sites at baseline (BL) and day 90 (D90) depending on whether pocket closure was achieved (n = 21) or not achieved (n = 19) post-treatment. Dotted black line indicates 5 mm. Between group comparisons refer to unpaired t-tests (ns: non-significant, ***p < 0.001), within-group comparisons refer to paired t-tests (†*p* < 0.001), graphs display mean ± 95% confidence interval. (**B**) Species-level Bray–Curtis based PCoA analysis of each group at baseline, statistics are PERMANOVA with raw p-value displayed. (**C**) Comparing Bray–Curtis similarity with baseline between groups, statistics refer to Mann–Whitney test, ns: non-significant. (**D**) Plotting the top 30 most abundant species at BL and D90 between groups, No; pocket closure not achieved, Yes; pocket closure achieved.

To establish whether microbial profiles impacted treatment response, an exploratory analysis was conducted and patients were grouped depending on whether ‘pocket closure’ was achieved in the sampled sites (defined as the conversion of a site measuring ≥ 5 mm to a site measuring ≤ 4 mm)^27,45^. Sites < 5 mm were sampled in two patients at baseline, and these patients were excluded from this analysis (Supplementary dataset 1). Evaluating pocket closure rates within the remaining sites (n = 40), 21 sites achieved pocket closure at day 90, whilst 19 sites did not. When comparing pocket depths at baseline between these two groups, no significant difference was observed indicating similar levels of starting disease severity (Fig. 6A). There were statistically significant reductions in pocket depth in both groups, albeit less pronounced in sites which did not achieve pocket closure (*p* < 0.001). To investigate the difference in the baseline microbiota of each group, a Bray–Curtis based principal coordinate analysis was performed (Fig. 6B). Despite differences in clinical outcomes, there was no significant difference in baseline microbial profiles of each group. Likewise, no difference was observed in Bray–Curtis similarity profiles (BL vs. D90), indicating similar microbial responses to NST (Fig. 6C). Comparing the relative abundance of species revealed no significant differences between groups at either timepoint (Fig. 6D), which was consistent for alpha-diversity indexes (data not shown). Additional exploratory analysis revealed no differences in baseline microbial profile or response among different demographic (sex, age) and behavioural subgroups (smoking status) within the current cohort (Supplementary Fig. 4).

### Inflammatory response, disease severity and response to treatment

**Figure 7:**
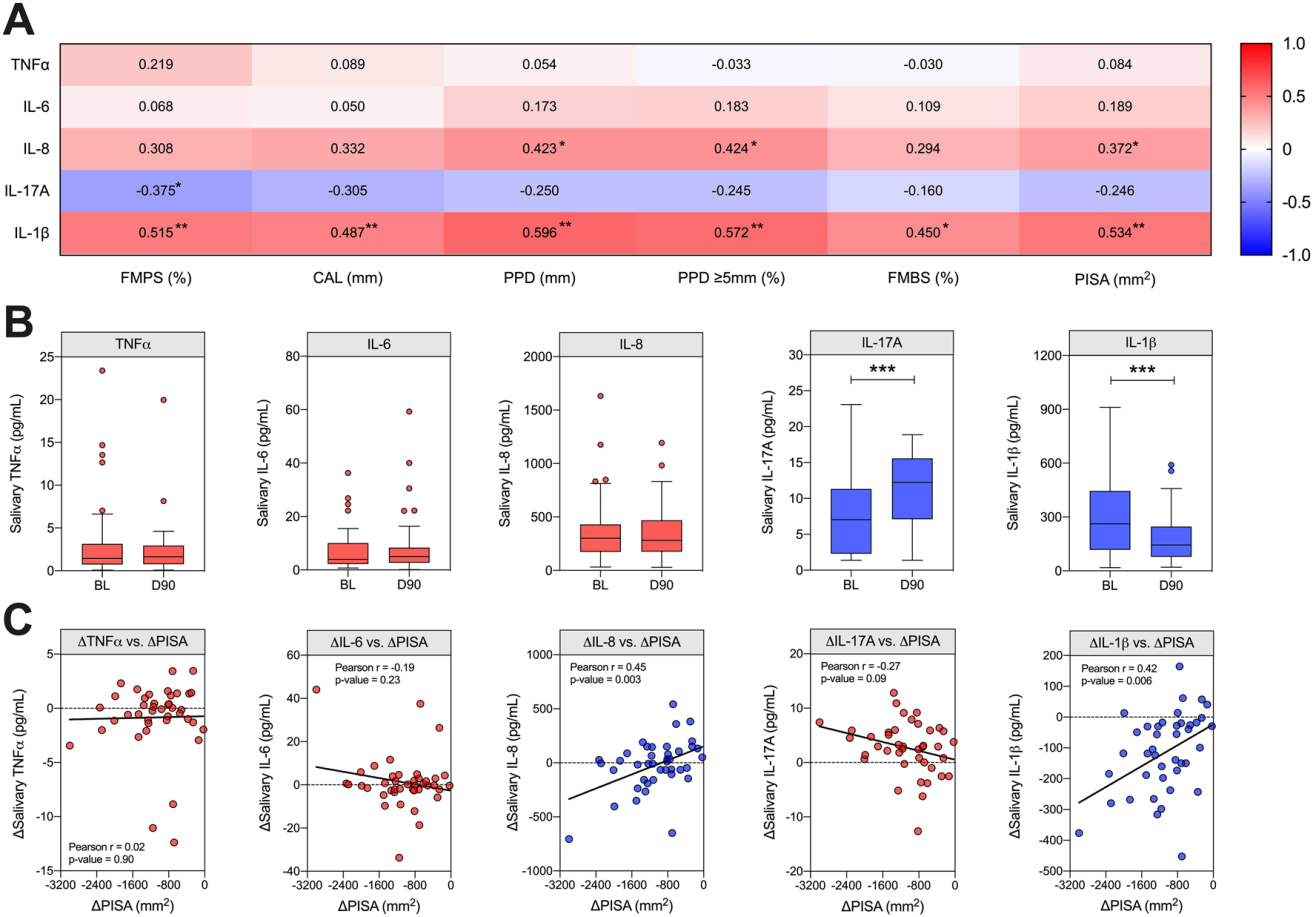
Levels of local inflammatory markers in saliva. (**A**) Correlating the levels of salivary inflammatory markers at baseline with clinical periodontal parameters, values represent Spearman-Rho correlation. P-values were corrected using FDR (5%) where *p(adjusted) < 0.05 and **p(adjusted) < 0.01. Confidence intervals are supplied in supplementary dataset 1 (**B**) Comparing changes in the levels of TNFα, IL-6, IL-8, IL-17A and IL-1β at baseline (BL) and day-90 (D90). Graphs are Tukey boxplots where horizontal line indicates the median and individual points indicate potential outliers. Statistics are Wilcoxon signed rank test, where ***p < 0.001. n = 41. (**C**) Pearson’s correlation between change in PISA and change in salivary cytokines, raw p-values are shown.

Given the key role of inflammation in periodontal pathogenesis, we next sought to investigate whether clinical and microbial improvements were accompanied by alterations in the local inflammatory response. At baseline, salivary IL-1β showed a positive association with all clinical parameters, including the PISA (r = 0.534, *p* < 0.001), which reflects the inflammatory burden of PD (Fig. 7A). A weak positive association was observed between salivary IL-8 and full-mouth pocket depth, proportion of deep periodontal pockets and the PISA (r = 0.423, 0.424, 0.372 respectfully), whilst a weak negative association was observed between IL-17A and plaque index (r = − 0.375, *p* = 0.049). Salivary TNFα and IL-6 showed no significant association with any periodontal clinical parameter. Additionally, no significant positive or negative association between genera or species abundance and salivary cytokines was observed at either timepoint following adjustment for multiple comparisons (Supplementary Table 4).

Following treatment (Fig. 7B), there was a notable reduction in salivary IL-1β (*p* < 0.001), and, somewhat surprisingly, an increase in the level of salivary IL-17A, albeit a quantitatively small change (*p* < 0.001). No significant longitudinal changes were observed for TNFα, IL-6 or IL-8. To further evaluate the relationship between clinical disease and local inflammation, we assessed correlations in the change in each cytokine against the change in PISA (Fig. 7C). The change in salivary IL-1β showed a positive correlation with the change in the PISA, matching its association with disease severity at baseline and reductions following treatment. Despite the lack of change at day 90, salivary IL-8 also showed a similar relationship (r = 0.45, *p* = 0.003), whereby those with the greatest reduction in PISA tended to show a reduction in salivary IL-8 following treatment, whilst patients with less pronounced changes appeared to show similar or increased levels compared with baseline.

## Discussion

Although previous studies have evaluated the microbiome following NSPT^46–48^, to our knowledge, this is the first study to simultaneously investigate longitudinal alterations in the subgingival plaque microbiome, local inflammatory markers and periodontal clinical parameters in the same patient cohort. Furthermore, this study has one of the largest cohorts for studying alterations in the subgingival plaque microbiome. In our study, we show a clear decrease in disease associated organisms in the subgingival microbiota after NSPT, which was accompanied by alterations in salivary cytokines. Although patients all showed positive clinical changes, only 47% obtained a PISA commensurate with periodontal health (130.33mm^2^)^49^—which is comparable with a myriad of published studies. Thus, defining how patients respond to non-surgical periodontal treatment—encompassing clinical, microbial and inflammatory markers is critical to rationally test and implement much needed NSPT adjuncts and novel therapeutics.

Previous studies comparing PH and PD have shown higher microbial diversity in the subgingival plaque of PD patients, suggesting that increased pocket depths, nutrient availability and/or immune impairment allow for growth of a more diverse biofilm community^16,50^. In line with this theory, we observed consistent reductions in diversity following treatment, matching with reduced pocket depths and clinical inflammation. Along with shifts in taxonomic diversity, we observed differential abundance of 42 genera and 61 species following NSPT, representing large-scale alterations in the microbiota composition. The majority of organisms that decreased following treatment were strict anaerobes (e.g. *Fusobacterium, Prevotella, Porphyromonas*), with increases found typically in facultative anaerobes or aerobes (e.g. *Kingella, Rothia, Actinomyces*). This trend indicates that in addition to physical biofilm disruption, clinical improvement likely drives environmental shifts which contribute to biofilm composition, such as increased oxygen availability (from decreased pocket depths) and the reduction of inflammation-related nutrient availability^51,52^.

Interestingly, we observed no significant microbiota differences between sites that did and did not achieve pocket closure, despite similar baseline disease severity. This study uniquely investigated the entire subgingival plaque microbiome factoring in pocket closure as a clinically relevant treatment outcome for patients receiving NSPT. We selected this outcome as the most clinically meaningful threshold which determines the need for further active treatment^27^. Similar data has been reported previously in a smaller cohort when PPD, CAL and BoP were used as indicators of treatment success^47^. An important observation to consider when interpreting the analysis from the current study is that even sites which did not achieve pocket closure did display significant, albeit less pronounced, clinical improvement following treatment. Future studies incorporating true ‘non-responders’ to NSPT would be useful to establish any causation between the baseline subgingival plaque microbiota and response to treatment.

Assessing longitudinal changes within the entire cohort, we observed a significant decrease in the levels of disease associated species following treatment, including *Fretibacterium fastidiosum*, *Fretibacterium feline* and *Filifactor alocis*. *Filifactor alocis* has been suggested as a biomarker for periodontitis and was proposed as a diagnostic marker organism^53,54^. In addition, *Fretibacterium* spp., including *F. fastidiosum*, have been associated with PD in different independent studies. Interestingly, red complex genera (*Porphyromonas*, *Treponema*, *Tannerella*) correlated positively with *Filifactor* and *Fretibacterium* at baseline in our study, which led to the formation of a clearly disease-associated cluster. After treatment, these associations were lost and a newly formed disease-associated network was formed, including genera that are abundant in health but increase in PD (e.g., *Fusobacterium* and *Prevotella*)^16^, along with *Treponema, Alloprevotella*, *Selenomonas*, *Parvimonas*, *Dialister* and *Catonella*. Whether groups of disease-associated organisms may persist to drive future disease recurrence remains unclear, and requires a longer study period with microbiological monitoring.

Positive associations provide a useful indicator of key interactions within the subgingival plaque that drive dysbiosis; although investigating negative associations between organisms is an often-overlooked concept in PD. Such associations may elucidate interactions which help maintain the biofilm in a eubiotic state and could be a useful starting point for identifying potential probiotics. In our study at day 90, we observed negative associations between *Rothia* and several PD-associated genera (*Selenomonas*, *Fusobacterium* and *Prevotella*), and a positive correlation between *Rothia* and *Actinomyces*. Both *Rothia* and *Actinoymces* are confirmed nitrate reducing genera^55,56^, and several strictly anaerobic PD-associated species have shown to be sensitive to oxidative stress and nitric oxide^57^. Thus, the possible role of nitric oxide and other antimicrobial components produced by these genera in the inhibition of PD-associated organisms and maintenance of biofilm eubiosis warrants further investigation.

In previous smaller studies assessing the subgingival plaque microbiome following NSPT, ungrouped genera such as *Dialister* and *Olsenella* decreased post-treatment, whilst *Rothia* and *Corynebacterium* increased ^47,48^, commensurate with our results. However, a greater number of microbial alterations were observed in the current study. For example, Shi et al*.,* demonstrated an increase in 4 genera and decrease in 8 genera across 12 patients following NSPT, whilst Chen et al*.,* included 19 patients and observed large patient-to-patient variation in microbial response. In our study of 42 patients, we identified consistent shifts in 42 genera and 61 species. One reason for these findings may be that in previous studies^48^, patients returned at varying timepoints (4–19 weeks) which may impact the stage of biofilm redevelopment and thus composition. In contrast, all patients returned at 90 days following treatment in this study, which is a recommended clinical follow-up period^58^. Taken together, a larger sample size and standardised follow-up period may help explain the greater number of microbial alterations found in the current study following treatment.

From an inflammatory perspective, this study highlights a clear association between salivary IL-1β and periodontal inflammation, supporting previous cross-sectional and longitudinal studies^25,59,60^. However, this result was not observed for other pro-inflammatory markers (TNFα, IL-6, IL-8). In general, salivary levels of TNFα and IL-6 are low^61,62^, and, similar to our findings, previous studies have failed to demonstrate a consistent relationship between salivary IL-8 and PD^63^. Saliva is a harsh environment containing mucins, bacteria and various proteolytic enzymes. Therefore, it is possible that low values of TNFα and IL-6 merely represents their ability to survive in this setting, rather than lack of production within gingival tissue.

To our surprise, we observed a significant increase in the levels of salivary IL-17A after treatment. Primarily produced by Th17 cells, IL-17A plays an important role in the host defence against external pathogens at mucosal sites^64^. Despite this function being co-aligned with the pathophysiology of PD (biofilm challenge in the periodontal pocket), in reality, the relationship between IL-17A and PD remains complex and unclear, with evidence to suggest both protective and destructive effects^65^. Previous cross-sectional studies assessing local production of IL-17A in the gingival crevicular fluid (GCF) and saliva have been largely incolclusive, with some studies reporting higher levels in PD^66,67^, whilst others find higher levels in PH^68,69^. Another study reported elevated levels in localised PD compared with generalised PD and PH, suggesting that IL-17A may peak in early gingival inflammation rather than established periodontitis^70^. In relation to our study, day 90 samples are reflective of a post-treatment state of stability, rather than total periodontal health. Given the findings by Liukkonen et al*.,* it is possible that treatment induces a shift back to an early inflammatory state, where IL-17A is a driving force. To further investigate this hypothesis, it would be beneficial for future studies to compare salivary IL-17A across a range of clinical states, including post-treatment PD, gingivitis and healthy patients.

Whilst the overall goal of this study was to identify longitudinal changes in clinical, microbial and immunological parameters post-treatment—certain limitations remain, and results should be interpreted in consideration of these factors. One such limitation is the lack of healthy control samples for a comparison with post treatment clinical health. Despite observing consistent shifts in known disease associated microbial and immunological markers^15,22,61,62,71^, future studies should investigate whether these resort back to a state comparable with periodontally healthy subjects. Another limitation is that patients were only followed-up at a single timepoint. Patients are evaluated three months following non-surgical treatment, and decisions made about future treatment plans^72^. Future studies could consider the impact of supportive treatment, repeated treatment, or adjunctive treatments on the microbiome, and whether the microbiome post NSPT could be used to guide decision making. Evaluating the microbiome at earlier timepoints would provide a much-needed indication of the early microbial recolonization pattern following biofilm disruption. Longer follow up evaluation (6 months—1 year) would establish whether shifts are maintained, and which (if any) could be used to predict disease recurrence.

In conclusion, we present the first longitudinal study of NSPT to evaluate local inflammatory cytokines and microbiome sequencing in the same patient cohort. Our data highlight large-scale subgingival community shifts following NSPT, consistent with the reformation of an ecosystem more compatible with PH. However, a tightly clustered network of disease-associated genera remained at day 90, underlining the resilience of a microbial core that may be partly responsible for the chronicity of the disease. From an inflammatory perspective, our study confirms the relationship between salivary IL-1β and PD, showing a close association with periodontal inflammation, and a proportional reduction following treatment. Collectively, this study shows that current treatment results in partial restoration of health but features of biofilm dysbiosis and inflammation remain. Means of modifying these parameters to improve clinical outcomes and reduce disease recurrence—that are not antibiotic dependent—are very much needed to improve periodontal treatment outcomes.

## Supplementary Information

Supplementary Information 1.

Supplementary Information 2.

## Data Availability

A supplementary dataset containing figure source data accompanies this manuscript. All sequencing reads are deposited in the NCBI Sequencing Read Archive (SRA) under BioProject PRJNA725103. Any further data is available upon reasonable request from the corresponding author.
