# Supplementary material for: Mechanical biofilm disruption causes microbial and immunological shifts in periodontitis patients
Source: Sci Rep. 2021 May 7;11:9796. doi: 10.1038/s41598-021-89002-z (PMC8105330; doi:10.1038/s41598-021-89002-z)
Supplement: Supplementary file 1 — Supplementary Information 1. [file 41598_2021_89002_MOESM1_ESM.docx]

Mechanical biofilm disruption causes microbial and immunological shifts in periodontitis patients

W. Johnston, B. T. Rosier, A. Artacho, M. Paterson, K. Piela, C. Delaney, J. L. Brown, G. Ramage, A. Mira, ^*^S. Culshaw

**Supplementary tables and figures**

**Supplementary table 1:** Baseline demographics of patients completing to the study (n=42). Data is presented as mean ± SD, n (%) or min-max.

| **Variable** | **n=42** |
| --- | --- |
| Age (years) | 50 ± 11 |
| Male, female (%) | 29, 71 |
| Smoking (never, former, current) (%) | 36, 40, 24 |
| Treatment visits (min-max) | 1 – 6 |
| Number of teeth | 26 ± 3 |

**Supplementary table 2:** Raw abundances, p-values and q-values for health and disease associated genus (n=42). Abundances are median (Q1, Q3).

| **Bacterial species** | **Abundance at baseline (%)** | **Abundance at day-90 (%)** | **p-value** | **FDR**  **q-value** | **Significance** |
| --- | --- | --- | --- | --- | --- |
| **Red complex^1^** | | | | | |
| *Porphyromonas_gingivalis* | 1.396  (0.491, 4.965) | 0.059  (0.000, 0.626) | 0.0002 | 0.0005 | *** |
| *Treponema_denticola* |  |  |  |  |  |
| *Tannerella_forsythia* |  |  |  |  |  |
| **^#^Orange complex^1^** | | | | | |
| *Prevotella_intermedia* | 12.440  (8.151, 18.52) | 3.287  (1.366, 12.970) | 0.0005 | 0.0009 | *** |
| *Prevotella_nigrescens* |  |  |  |  |  |
| *Fusobacterium_nucleatum* |  |  |  |  |  |
| *Fusobacterium_periodonticum* |  |  |  |  |  |
| *Parvimonas_micra* |  |  |  |  |  |
| *Campylobacter_gracilis* |  |  |  |  |  |
| *Streptococcus_constellatus* |  |  |  |  |  |
| *Campylobacter_showae* |  |  |  |  |  |
| **Green complex^1^** | | | | | |
| *Aggregatibacter_actinomycetemcomitans* | 0.999  (0.456, 3.534) | 1.079  (0.360, 3.049) | 0.8430 | 0.8430 | ns |
| *Eikenella_corrodens* |  |  |  |  |  |
| *Capnocytophaga_ochracea* |  |  |  |  |  |
| *Capnacytophaga_gingivalis* |  |  |  |  |  |
| *Capnocytophaga_sputigena* |  |  |  |  |  |
| **Purple complex^1^** | | | | | |
| *Actinomyces_odontolyticus* | 1.160  (0.503, 2.761) | 0.790  (0.329, 1.716) | 0.1249 | 0.1458 | ns |
| *Veillonella_parvula* |  |  |  |  |  |
| **Blue complex^1^** | | | | | |
| *Actinomyces_cardiffensis* | 2.162  (1.023, 4.040) | 5.768  (1.417, 10.560) | 0.0038 | 0.0053 | ** |
| *Actinomyces_israelii* |  |  |  |  |  |
| *Actinomyces_graevenitzii* |  |  |  |  |  |
| *Actinomyces_meyeri* |  |  |  |  |  |
| *Actinomyces_hongkongensis* |  |  |  |  |  |
| *Actinomyces_oris* |  |  |  |  |  |
| *Actinomyces_gerencseriae* |  |  |  |  |  |
| *Actinomyces_NA* |  |  |  |  |  |
| *Actinomyces_naeslundii* |  |  |  |  |  |
| *Actinomyces_massiliensis* |  |  |  |  |  |
| *Actinomyces_georgiae* |  |  |  |  |  |
| **Yellow complex^1^** | | | | | |
| *Streptococcus_NA* | 7.832  (5.702, 12.700) | 15.460  (10.280, 19.910) | 0.00004 | 0.0002 | *** |
| *Streptococcus_cristatus* |  |  |  |  |  |
| *Streptococcus_anginosus* |  |  |  |  |  |
| *Streptococcus_gordonii* |  |  |  |  |  |
| *Streptococcus_mutans* |  |  |  |  |  |
| *Streptoccus_oralis* |  |  |  |  |  |
| *Streptococcus_parasanguinis* |  |  |  |  |  |
| *Streptococcus_sobrinus* |  |  |  |  |  |
| *Streptococcus_salivarius* |  |  |  |  |  |
| *Streptococcus_sinensis* |  |  |  |  |  |
| *Streptococcus_massiliensis* |  |  |  |  |  |
| **Novel disease associated species^2^** | | | | | |
| *Treponema_medium* | 8.472  (3.296, 13.610) | 1.542  (0.055, 5.057) | 0.00004 | 0.0002 | *** |
| *Peptostreptococcus_stomatis* |  |  |  |  |  |
| *Prevotella_denticola* |  |  |  |  |  |
| *Mogibacterium_timidum* |  |  |  |  |  |
| *Filifactor_alocis* |  |  |  |  |  |
| *Selenomonas_sputigena* |  |  |  |  |  |
| *Alloprevotella_tannerae* |  |  |  |  |  |
| *Anaeroglobus_geminatus* |  |  |  |  |  |
| *Fretibacterium_fastidiosum* |  |  |  |  |  |
| *Porphyromonas_endodontalis* |  |  |  |  |  |
| *Treponema_vincentii* |  |  |  |  |  |
| *Treponema_lecithinolyticum* |  |  |  |  |  |
| *Dialister_pneumosintes* |  |  |  |  |  |

^1^As outlined in Socransky *et al.,* (1998)

^2^As outlined in Perez-Chaparro *et al.,* (2014), excluding those previously classified^1^

*q<0.05, **q<0.01 FDR corrected Wilcoxon signed rank test.

**^#^***Eubacterium nodatum* and *Campylobacter rectus* from the orange complex were not classified at species-level in our cohort.

**Supplementary table 3:** Spearman-Rho correlation between alpha diversity indexes, genera and species with pocket depth of sampled teeth at baseline and following treatment (day 90). For genera and species correlations, only displaying p(unadjusted)<0.05.

| **Organism** | **Spearman R** | **p-value** | **Adjusted p-value** |
| --- | --- | --- | --- |
| Alpha diversity (baseline) | | | |
| Shannon index | 0.403 | 0.008 | - |
| Chao1 index | 0.312 | 0.044 | - |
| ACE index | 0.305 | 0.049 | - |
| Alpha diversity (day 90) | | | |
| Shannon index | 0.194 | 0.217 | - |
| Chao1 index | 0.180 | 0.255 | - |
| ACE index | 0.204 | 0.195 | - |
| Genus-level (baseline) | | | |
| *Saccharimonadales_NA* | 0.383 | 0.012 | 0.415 |
| *Lentimicrobiaceae_NA* | 0.340 | 0.028 | 0.415 |
| *Olsenella* | 0.308 | 0.0475 | 0.415 |
| *Alloprevotella* | 0.306 | 0.0488 | 0.415 |
| Species-level (baseline) | | | |
| *Tannerella_NA* | 0.415 | 0.006 | 0.382 |
| *Alloprevotella_tannerae* | 0.337 | 0.029 | 0.451 |
| *Selenomonas_sputigena* | 0.309 | 0.047 | 0.580 |
| Genus-level (day 90) | | | |
| *Treponema* | 0.508 | 0.0006 | 0.019 |
| *Prevotella* | 0.477 | 0.001 | 0.019 |
| *Parvimonas* | 0.446 | 0.003 | 0.020 |
| *Alloprevotella* | 0.444 | 0.003 | 0.020 |
| *Dialister* | 0.440 | 0.004 | 0.020 |
| *Clostridiales Family_XIII_NA* | 0.430 | 0.005 | 0.022 |
| *Tannerella* | 0.327 | 0.034 | 0.097 |
| *Campylobacter* | 0.323 | 0.037 | 0.097 |
| *Corynebacterium* | -0.470 | 0.002 | 0.019 |
| *Granulicatella* | -0.403 | 0.008 | 0.035 |
| *Kingella* | -0.390 | 0.011 | 0.040 |
| *Leptotrichia* | -0.334 | 0.031 | 0.097 |
| *Bergeyella* | -0.323 | 0.037 | 0.097 |
| Species-level (day 90) | | | |
| *Parvimonas_micra* | 0.446 | 0.003 | 0.061 |
| *Dialister_invisus* | 0.418 | 0.006 | 0.061 |
| *Selenomonas_sputigena* | 0.397 | 0.009 | 0.063 |
| *Prevotella_NA* | 0.381 | 0.013 | 0.067 |
| *Fusobacterium_nucleatum* | 0.369 | 0.016 | 0.067 |
| *Selenomonas_NA* | 0.367 | 0.017 | 0.067 |
| *Campylobacter_gracilis* | 0.364 | 0.018 | 0.067 |
| *Corynebacterium_matruchotii* | -0.477 | 0.001 | 0.058 |
| *Granulicatella_NA* | -0.403 | 0.008 | 0.063 |
| *Corynebacterium_durum* | -0.371 | 0.016 | 0.067 |
| *Cardiobacterium_hominis* | -0.351 | 0.023 | 0.077 |
| *Leptotrichia_NA* | -0.317 | 0.041 | 0.128 |
| *Bergeyella_NA* | -0.310 | 0.046 | 0.134 |

**Supplementary table 4:** Spearman-Rho correlation between relative abundance of genera and species with salivary inflammatory cytokines at baseline and day 90. Displaying p(unadjusted)<0.05, data displayed as Spearman R, raw p-value (adjusted p-value).

| **Bacteria** | **TNFα** | **IL-6** | **IL-8** | **IL-17A** | **IL-1β** |
| --- | --- | --- | --- | --- | --- |
| **Genus-level (baseline)** | | | | | |
| *Rikenellaceae_RC9* | 0.46  p=0.003 (0.12) | - | - | - | - |
| *Paludibacteraceae_F0058* | 0.31  p=0.047 (0.40) | - | - | - | - |
| *Lachnoanaerobaculum* | -0.32  p=0.04 (0.40) | - | - | - | - |
| *Kingella* | -0.36  p=0.02 (0.40) | - | - | - | -0.32  p=0.04 (0.39) |
| *Fusobacterium* | - | - | -0.34  p=0.032 (0.94) | - | -0.35  p=0.02 (0.34) |
| *Filifactor* | - | - | - | -0.33  p=0.04 (0.70) | - |
| *Saccharimonadaceae_NA* | - | - | - | - | 0.39  p=0.01 (0.27) |
| *Alloprevotella* | - | - | - | - | 0.38  p=0.01 (0.27) |
| *Saccharimonadales_NA* | - | - | - | - | 0.31  p=0.048 (0.39) |
| **Species-level (baseline)** | | | | | |
| *Kingella_oralis* | -0.39  p=0.01 (0.32) | - | - | 0.31  p=0.04 (0.66) | -0.33  p=0.03 (0.34) |
| *Treponema_denticola* | 0.37  p=0.02 (0.32) | - | - | - | - |
| *Capnocytophaga_ochracea* | -0.36  p=0.02 (0.32) | - | - | - | - |
| *Capnocytophaga_gingivalis* | - | -0.37  p=0.02 (0.98) | - | - | - |
| *Corynebacterium_matruchotii* | - | -0.32  p=0.04 (0.98) | - | - | - |
| *Eikenella_corrodens* | - | - | -0.32  p=0.04 (0.87) | - | - |
| *Selenomonas_NA* | - | - | 0.32  p=0.04 (0.87) | - | 0.36  p=0.02 (0.34) |
| *Rothia_dentocariosa* | - | - | - | 0.35  p=0.03 (0.66) | - |
| *Filifactor_alocis* | - | - | - | -0.34  p=0.03 (0.66) | - |
| *Fusobacterium_nucleatum* | - | - | - | - | -0.35  p=0.03 (0.34) |
| *Alloprevotella_tannerae* | - | - | - | - | 0.35  p=0.03 (0.34) |
| *Selenomonas_sputigena* | - | - | - | - | 0.33  p=0.03 (0.34) |
| **Genus-level (day 90)** | | | | | |
| *Parvimonas* | -0.36  p=0.02 (0.61) | - | - | - | - |
| *Tannerella* | -0.31  p=0.047 (0.64) | - | - | - | - |
| *Eikenella* | - | -0.37  p=0.02 (0.25) | - | - | - |
| *Bergeyella* | - | -0.37  p=0.02 (0.25) | - | - | - |
| *Saccharimonadaceae_NA* | - | - | 0.38  p=0.01 (0.43) | - | - |
| *Actinomycetaceae_F0332* | - | - | - | - | 0.35  p=0.03 (0.46) |
| *Selenomonas* | - | - | - | - | 0.33  p=0.04 (0.46) |
| **Species-level (day 90)** | | | | | |
| *Parvimonas_micra* | -0.36  p=0.02 (0.66) | - | - | - | - |
| *Capnocytophaga_ochracea* | - | - | -0.47  p=0.002 (0.07) | - | - |
| *Haemophilus_NA* | - | - | -0.37  p=0.02 (0.24) | - | - |
| *Eikenella_NA* | - | - | - | -0.31  p=0.048 (0.91) | - |
| *Selenomonas_NA* | - | - | - | - | 0.42  p=0.007 (0.25) |
| *Selenomonas_sputigena* | - | - | - | - | 0.36  p=0.02 (0.34) |

**Supplementary figure 1: Significantly different genera (A) and species (B) following treatment.** Displaying Log_2_ fold change (mean at day 90/mean at baseline) vs. mean abundance at baseline. Only genera and species which met the abundance threshold were included for statistical analysis (see methods section). In total, 42 genera differed significantly following treatment, with 34 decreasing and 8 increasing in abundance. The genera *Centipeda* (labelled) significantly reduced following treatment but displayed a positive fold-change due to outliers. At species-level, 61 differed significantly with 46 decreasing and 15 increasing in abundance. Statistics refer to Wilcoxon signed rank tests, adjusted for multiple comparisons using FDR (5%). Horizontal line indicates p(adjusted)=0.05. n=42.

**Supplementary figure 2: Genera and species composition**. Composition of baseline (BL) and day-90 (D90) samples determined by 16S rRNA sequencing, displaying the top 20 most abundant genera (A) and top 30 most abundant species (B).

**Supplementary figure 3: Correlation circle plots for association networks.** Association between genera and species are obtained based on their projection onto a correlation circle plot derived from a principal component analysis, as described by González *et al.,* (45). This was performed at genus-level at baseline (A) and day 90 (B), and species-level at baseline (C) and day 90 (D). Associations were complemented with Spearman-Rho correlation coefficients, see **Supplementary dataset 1**.


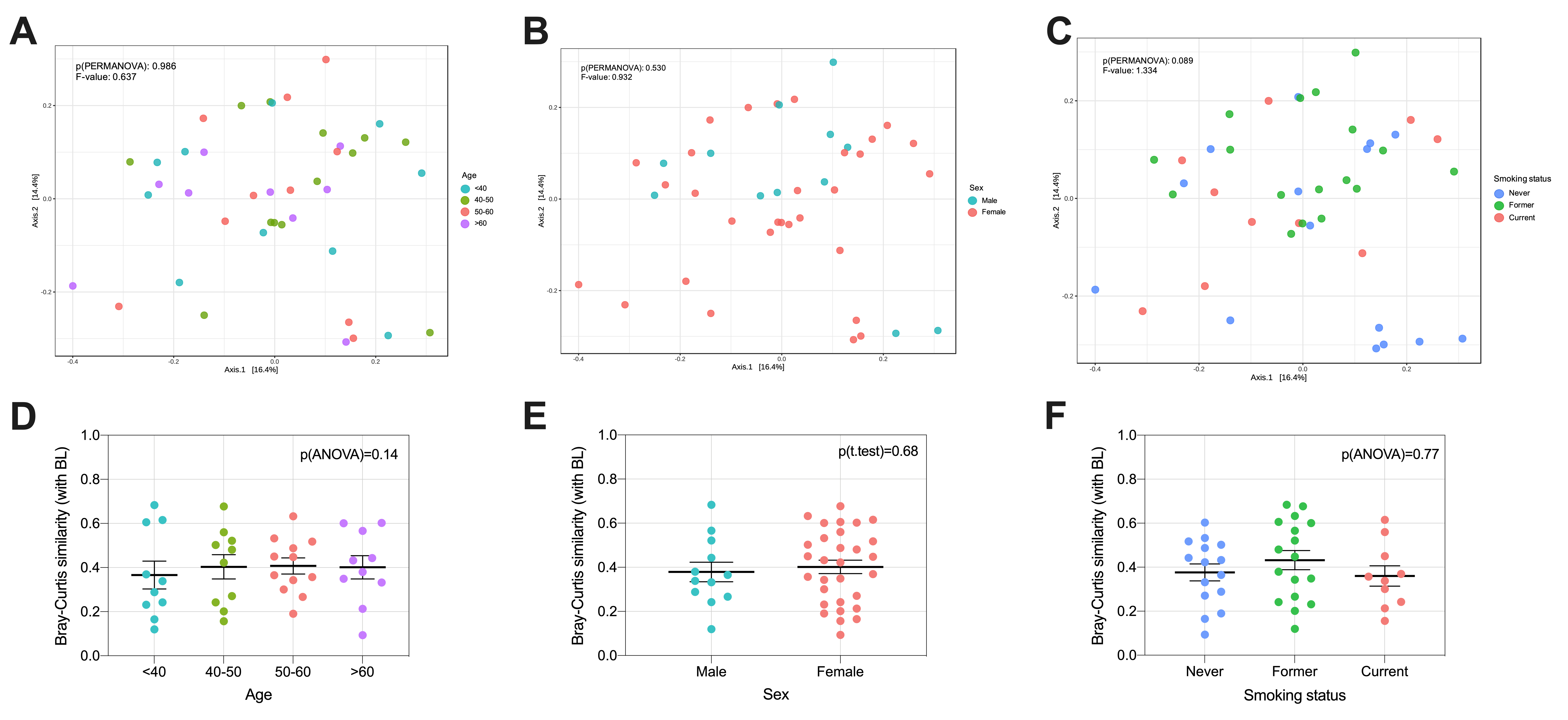


**Supplementary figure 4: Associations between demographic and behavioural variables and the subgingival plaque microbiome.** PCoA analysis of the subgingival plaque microbiome at baseline across age (A), sex (B) and smoking status (C) categories. Statistics are PERMANOVA, with p-values displayed on graph. Comparing Bray-Curtis similarity with baseline across age (D), sex (E) and smoking status (F) categories, statistics refer to ANOVA with Tukey post-hoc (D, F) or unpaired t-test (E).
